# Gender Differences in Motor Skills of the Overarm Throw

**DOI:** 10.3389/fpsyg.2017.00212

**Published:** 2017-02-17

**Authors:** Michael Gromeier, Dirk Koester, Thomas Schack

**Affiliations:** ^1^Faculty of Psychology and Sport Science, Neurocognition and Action – “Biomechanics” Research Group, Bielefeld UniversityBielefeld, Germany; ^2^Center of Excellence “Cognitive Interaction Technology”, Bielefeld UniversityBielefeld, Germany; ^3^Research Institute for Cognition and Robotics – CoR-Lab, Bielefeld UniversityBielefeld, Germany

**Keywords:** overhead throwing, throwing accuracy, throwing performance, motor skill development, handball

## Abstract

In this cross-sectional study, the qualitative and quantitative throwing performance of male and female athletes (6 to 16 years of age) was analyzed. The goal of this study was to assess whether there were gender based qualitative and quantitative differences in throwing performance of young athletes, throughout three different age bands (childhood, pubescence, and adolescence). Furthermore, we explored whether all components of the throwing movement are equally affected by gender differences. Focus was placed on five essential components of action: trunk, forearm, humerus, stepping, and backswing. Therefore, children and adolescents (*N* = 96) were invited to throw three times from three different distances, while aiming at a target placed at shoulder height. The participants were aspiring athletes, competitive in the sport handball. For analyzing the quality of movement the component approach of Halverson and Roberton (1984) was used. The throwing accuracy was noted and used to evaluate the quantitative performance of the throwing movement. Throughout three different age bands, no statistically significant difference was found between genders in throwing accuracy, i.e., quantitative performance. Regarding the qualitative evaluation of the throwing movement, male and female athletes differed significantly. The component approach yielded higher scores for male than for female participants. As expected, with increasing age qualitative and quantitative performance of male and female athletes improved. These results suggest that there are gender-specific differences in qualitative throwing performance, but not necessarily in quantitative throwing performance. Exploration shows that differences in the qualitative throwing performance were seen in specific components of action. Male and female athletes demonstrated similar movement patterns in humerus and forearm actions, but differed in trunk, stepping, and backswing actions.

## Introduction

Throwing is a basic and complex motor skill. It is said to be, “one of the most difficult fundamental motor skill[s] for children and adults and its acquisition requires coordination of the whole body” (Hamilton and Tate, 2002, p. 49). This motor skill is an important part of the integrative (non-specific) concept of team-ball games according to the game implicit learning model of Roth et al. (2002). Under this concept, sport games are grouped according to rebound games, goal-scoring games and throwing games (invasion games). Among throwing games, the technically correct throwing movement is relevant for, and comparable to many sport disciplines such as handball; yet it is also fundamental for learning the process of the javelin throw. The importance of high level sport-specific motor skills in team sports have already been formulated by Schack and Bar-Eli (2007, S. 62). “The success of an individual athlete or a team is highly dependent on how well the essential techniques of the sport are applied and mastered.” Typical handball specific situations in which the overarm throwing movement comes into effect are 7-m throws, free-throws, backcourt throws, and the initiation of fast breaks (Kolodziej, 2010). The overarm throwing movement is also a form related to the jump shot, which is the most important and most commonly used throwing technique in handball (Wiener, 2011). Hence, this motor skill is an important part of school and university education, as well as belonging to club sport.

The accuracy and the velocity of throwing are often used to investigate the differences in quantitative characteristics of throwing movements. Gender differences in throwing velocity have been identified by Roberton and Konczak (2001). From the ages of 6 to 13, boys performed better compared with girls of the same age. In addition, Roberton and Konczak (2001) see an increasing amount of difference in the throwing performance. At a certain level of development only boys improve their skills, while the girls stagnated at their level, or became worse. A large meta-analysis of Thomas and French (1985) recognizes gender differences in throwing accuracy, and the velocity already at the age of three. Boys exceed girls in throwing velocity from 4 to 7 years of age and throwing distance at 2 to 4 years of age. Morris et al. (1982) examined the influence of age and gender on the performance of seven basic motor skills. Two of them (tennis ball throw for distance, and softball throw for distance) were related to throwing movements. Regarding throwing velocity and throwing accuracy, boys at the age of 3 to 6 years were superior to girls of the same age. Similar results are described by Vogt (1978). His studies show significantly better performance in the throwing accuracy in favor of boys between 5 and 6 years of age. Within the quantitative characteristics of throwing movement reported studies (Vogt, 1978; Morris et al., 1982; Thomas and French, 1985; Roberton and Konczak, 2001) consistently emphasize better performances of male novices.

The quantitative characteristics of throwing movements of experts and beginners in handball were analyzed by van den Tillaar and Ettema (2004). They evaluated Norwegian handball players at the age of 20 to 24 years. The task was to throw a ball as hard as possible to a 50 cm × 50 cm target. van den Tillaar and Ettema (2004) found gender differences in the throwing velocity in favor of men, when compared to women (23.2 m⋅s^-1^ for men and 19.2 m⋅s^-1^ for women). The studies of Rousanoglou et al. (2015) clarify quantitative differences between experts and amateurs. Amateurs performed with significantly lower throwing velocity and worse throwing accuracy (*p* < 0.001). Gorostiaga et al. (2005) found similar results when comparing two handball male teams: expert team, one of the worlds leading team and amateur team playing in the Spanish National Second Division. Experts showed significantly higher values in velocity of throwing without stepping (23,8 ± 1.9 m⋅s^-1^ vs. 21,8 ± 1.6 m⋅s^-1^, *p* < 0.05) and three-step rhythm (25,3 ± 2.2 m⋅s^-1^ vs. 22,9 ± 1.4 m⋅s^-1^, *p* < 0.05) than amateurs using the same approach. Rivilla-García et al. (2010) found significant differences in velocity between senior players and U-18 players in different throwing situations: (1) heavy medicine ball throw, (2) light medicine ball throw, (3) throwing velocity without opposition, and (4) throwing velocity with opposition. Senior players were found to perform significantly better than the U-18 players in all four throwing situations (*p* < 0.001; *t_1_* = 6.958; *t_2_* = 8.244; *t_3_* = 8.059; *t_4_* = 5.399; *df* = 92). Studies of quantitative characteristics of throwing within active handball players showed a usually better performance of male athletes than from comparable female athletes.

Qualitative assessments of throwing movement are commonly documented by photographs or videos in controlled or game settings, and were often based on the component analysis of Halverson and Roberton (1984). They designed a component approach, which analyzed the development of the full body movement. Therefore the full body movement was segmented in partial body movement (see **Table 1**). A partial body movement is one particular component or joint action of the body (Goodway and Lorson, 2008). This approach allows evaluating relevant nodes of the throwing movement and a separated classification. The component approach is the most extensive and the most used analysis of throwing movements, (e.g., Roberton and Konczak, 2001; Barrett and Burton, 2002; Hamilton and Tate, 2002; Langendorfer and Roberton, 2002; Ehl et al., 2005; Goodway and Lorson, 2008). A more detailed characteristic is described in Halverson and Roberton (1984).

**Table 1:** Component approach.

Component	Grade	Characteristic
(1) Trunk	T0	Varying movement in trunk action
	T1	No trunk action or forward-flexion
	T2	Upper trunk rotation or block rotation
	T3	Differentiated rotation

(2) Humerus	H0	Varying movement in humerus action
	H1	Humerus oblique
	H2	Humerus aligned but independent
	H3	Humerus lags

(3) Forearm	F0	Varying movement in forearm action
	F1	No forearm lag
	F2	Forearm lag
	F3	Delayed forearm lag

(4) Stepping	S0	Varying movement in stepping
	S1	No step
	S2	Ipsilateral step
	S3	Contralateral, long step

(5) Backswing	B0	Varying movement in backswing
	B1	Backswing behind or beside the head
	B2	Backswing behind or beside the head Insufficient backward extension
	B3	Nearly complete backward extension


*The five components according to the modified component approach (Halverson and Roberton, 1984) and the main developmentally characteristic levels (T, trunk; H, humerus; F, forearm; S, stepping; B, backswing). Grades from worst (0) to optimal (3) movements. Modified Component Approach by Halverson and Roberton (1984).*

Published studies refer to gender differences in component levels within each age group. For example, Lorson et al. (2013) found gender differences among adolescent and young adults; Goodway and Lorson (2008) recognized qualitative gender differences in game situations of children aged 6 to 8 years. They found significant (*p* < 0.017) gender differences for step, trunk and forearm components. In a 3-year longitudinal study, Nelson et al. (1991) investigate 100 children from 5 years of age, and the gender differences in qualitative and quantitative terms. Nelson et al. (1991) also reviewed the quality of trunk and the stepping actions and found differences in the trunk and the step in favor of the boys. Menzel (1999) analyzes highly skilled female javelin thrower to identify changes of individual movement patterns in the javelin throw. He found different movement patterns in the course characteristics of the knee angle and in the angles which define the trunk movement, which is associated with performance level. Results of previous studies mostly suggest that the qualitative performance of females developed slower than their male counterparts.

### Instruction to Investigate Kinematics of Throwing

Different instructions were used to determine quantitative characteristics such as velocity, hardness or accuracy, i.e., “throw hard” (Halverson et al., 1982, p. 199; Roberton and Konczak, 2001, p. 94), “each participant performed maximal-effort throws” (Lorson et al., 2013, p. 240), “maximum velocity” (Karadenizlil et al., 2014, p. 21), “throw normally” (Etnyre, 1998, p. 1211) or “throw the ball softly, increasing the speed until […] near-maximum speed(s)” (Intermill and Husak, 1984). van den Tillaar and Ettema (2006) compared the velocity and accuracy outcome of the overarm throw by using different instructions. They found, that “no trade-off between speed and accuracy was found for novices [*…*] or experts […] and accuracy did not change by instruction” (van den Tillaar and Ettema, 2006, p. 503). Contrary to the quantitative measurements, the instructions are relevant for qualitative measurements (Etnyre, 1998). Burton and Rodgerson (2003) denote most studies as non-functional when people are instructed to throw as hard as possible without paying attention to the throwing accuracy (Schott, 2010). Therefore, in the present study throwing accuracy and velocity are combined in one task.

Summarized gender-specific differences can be seen in throwing velocity, throwing accuracy, and movement quality characteristics. In addition, the motor performance increases with age and training experience. Most of the studies that analyzed the throwing movements refer to the novices, (e.g., Halverson et al., 1982; Bingham et al., 1989; Hamilton and Tate, 2002; Lorson et al., 2013). A comparison of experts and novices was made by van den Tillaar and Ettema (2003), Gorostiaga et al. (2005), Rivilla-García et al. (2010), and Rousanoglou et al. (2015). But in the latter studies, only velocity and accuracy were analyzed. The relationship of quantitative and qualitative throwing performance, age, and gender among athletes was not evaluated. The present cross-sectional study analyzes the qualitative and quantitative throwing performance of male and female athletes throughout three age bands.

The goal of this study was to assess whether there were gender based qualitative and quantitative differences in throwing performance of young athletes. It is hypothesized that the qualitative and quantitative performance of the overarm throwing movement differs between male and female athletes. It was predicted that the quantitative and the qualitative performance of the throwing movement of male athletes would be better than female athletes. With focus on five essential components of action: trunk, forearm, humerus, stepping, and backswing, we furthermore aimed to explore whether all components are equally affected by gender differences.

## Materials and Methods

### Participants

In this cross-sectional study, 96 skilled participants between the age of 6 and16 years participated (*M*_age_ = 11.93, *SD*_age_ = 3.190; 37 female; 59 male). At the time of examination, all athletes were members of sports clubs and active handball players. The handball teams were competitive, sport-oriented clubs. Male and female athletes played in the highest leagues of their age groups. Throughout the motor testing all participants were healthy and in good conditions. To conduct our study, written, full informed consent of the parents (or, alternatively, of the adult participants) were obtained, in accordance with the latest revision of the Helsinki Declaration of 1975 (World Medical Association [WMA], 2013). Our study was also approved by the ethics committee (“Ethik-Kommission”) at Bielefeld University and adhered to the ethical standards of the latest revision of the Declaration of Helsinki. Additionally, and also in accordance with the Helsinki Declaration of 1975 (World Medical Association [WMA], 2013), was the gathering of personal information such as body size, sex, age, handedness, club membership, training age, and training frequency per week. In the childhood the handball athletes already had a high amount of training years (*M* = 3.5, *SD* = 1.94) and training hours per week (*M* = 2.1, *SD* = 0.969). The average training age of all handball athletes was 5.8 years, and the average training frequency was 5.2 h per week. For data analysis, we formed three different age bands according to levels of development in the motor ontogenesis (Meinel and Schnabel, 1998, p. 240): (a) childhood (age of female 7 – 12, age of male 7 – 13), (b) pubescence, (age of female 12,1 – 14; age of male 13,1 – 14,5), (c) adolescence (age of female 14,15 – 16, age of male 14,6 – 16.

### Task and Procedure

**FIGURE 1:**
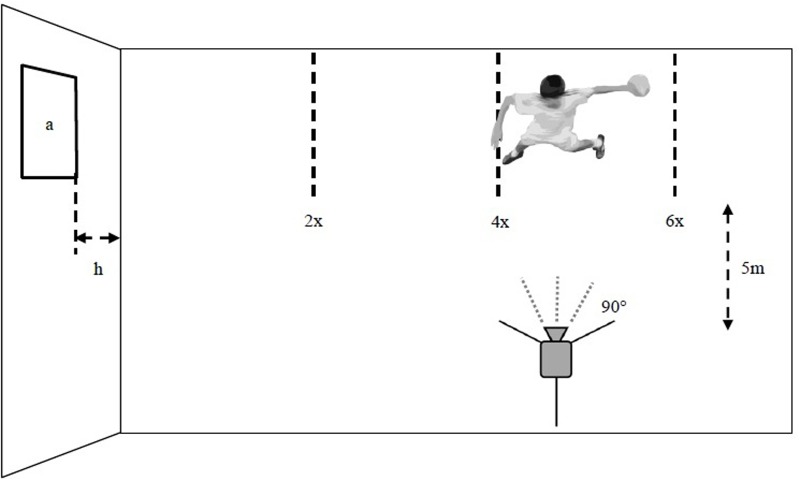
**Schematic drawing of the experimental setup of the motor testing and movement analysis from a bird’s-eye view.** (x = body height, target size (a) 75 cm × 75 cm in shoulder height (h), camera at a 90° angle at a distance of 5 m to the participant).

After a warm-up of about 15 min, which consisted of different exercises to prepare throwing movements, the participants were individually tested. The participants who were still being tested or have already been tested took part in further training. If possible, a visual delimination had been included in order to avoid participants potentially distracting each other. To assess the quality and the quantity of movement, the throwing accuracy and the throwing velocity was combined in one task. The instruction was to ‘throw as hard and accurate as possible.’ After the movement task was explained, the participants threw out of a 2.5 to 2.5 m tall corridor. The participants aimed at a square target (75 cm × 75 cm) with a self-selected ball. The choice between different ball sizes was in accordance to the quality standard of the International Handball Federation (00, 0, 1, 2, and 3) and also a tennis ball. It was important that the examiner made sure that the participants were able to hold the ball safely with one hand. The target was placed at shoulder height, and the participants threw three times from three different distances, for a combined total of nine throws. The distances of the participant’s throws were determined by multiplying their body-height by two, four, and six (see **Figure 1**). The movement testing took 5 min. The test set-up was similar to handball in that the throwing distances are in the range of the free-throw line (7-m line). The gradations matched the height of the participants which allowed for individual testing. This enabled more precise statements about throwing performances, and compensated for possible deficits, such as strength requirements.

The movement analysis of each trial was recorded with a Sony HDR-CX410VE wide angle lens camera with a high-speed 1/10000 shutter. A side view placed at a 90° angle from a distance of 5 m was used.

### Data Collection

To analyze the quantitative performance, any attempt which hit the target or which touched the mark of the target was considered as being successful. The proof of reliability for the results from the three different distances (see **Figure 1**) revealed an internal consistency of α = 0.657. The item-scale-statistic shows a low positive discriminatory power for the first distance (two times body height). In addition, the reliability improved (α = 0.741), if item deleted. Therefore the quantitative performance measured from two times body height was excluded and not used in the data analysis.

The qualitative performance was calculated as the mean of three independent expert ratings. At the time of the study, the experts were current handball coaches with an A- or B-license, and additionally, one expert had been a former international handball player. By using the video recording the experts had made evaluations in all five components (trunk, humerus, forearm, stepping, and backswing) for each participant. Based on Robertson’s component approach (Halverson and Roberton, 1984; see **Table 1**) for each trial the participant’s movement was computed for the different components in grades from worst (0) to optimal (3) movement. This resulted in a performance score for each component. Subsequently, the reliability of these ratings were calculated. Using Cronbach’s alpha, the internal consistency of the observer agreement was assessed. The internal consistency of the observer is in an acceptable and good range (α = 0.782 (Expert I), α = 0.795 (Expert II), and α = 0.746 (Expert III)). Then the performance scores of an expert were grouped in one qualitative performance score. One qualitative performance score represents an expert’s rating across all five components. So, for each participant there exist three qualitative performance scores. Finally, the qualitative performance was formed by all three qualitative performance scores. The evaluation of the qualitative movement refers to this qualitative performance.

### Data Analysis

In this cross sectional study, a 3 × 2 age (childhood, pubescence, and adolescence) and gender (male and female) one-way analysis of variance (ANOVA) and a significance level of 0.05 was used to identify effects of age, effects of gender, and interaction effects in quantitative performance of the throwing movement. For *post hoc* analysis, *t*-tests for independent samples were conducted. A Bonferroni Holm correction was employed to adjust the significance level in order to locate significant differences among age bands (Holm, 1979).

## Results

### Quantitative Characteristics of Throwing Movement

**FIGURE 2:**
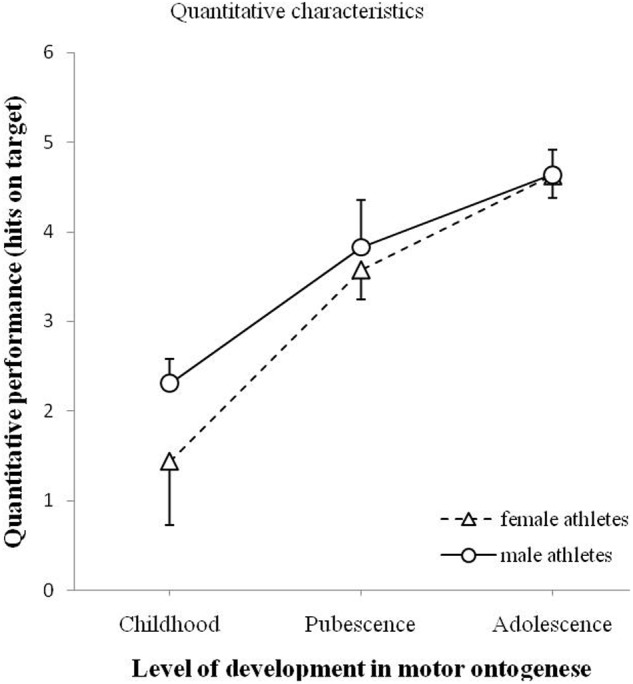
**Quantitative performances (minimum 0 – maximum 6 hits) for male and female athletes as a function of levels of development of motor ontogenesis.** The solid line (circles) illustrates the quantitative performance of male athletes in handball. The dashed line (triangles) illustrates the quantitative performance of female athletes in handball. Standard error marked by the error bars.

**Figure 2** illustrates the relationship between the quantitative performance of male and female athletes, and the level of development in motor ontogenesis (childhood, pubescence, and adolescence). To compare the effect of gender and the effect of age on quantitative performance, an ANOVA was used. ANOVA was also used to compare the interaction effects between gender and age on quantitative performance. The analysis showed no significant effect of gender on quantitative performance, *F*(1,90) = 1.207, *p* = 0.275. The analysis yielded a significant effect of age on quantitative performance, *F*(2,90) = 23.848, *p* < 0.001. The interaction effect was not significant, *F*(1,90) = 0.582, *p* = 0.561.

To follow-up the main effect of age, separate *t*-tests were performed (using Bonferroni Holm correction). *T*-tests for independent samples showed a significant age effect on quantitative performance between childhood and pubescence, *t*(67) = -3.738, *p* < 0.001, between pubescence and adolescence, *t*(38.67) = -2.586, *p* = 0.014. Consistently, adolescence yielded also better quantitative performances than childhood, *t*(69.633) = -7.782, *p* < 0.001.

### Qualitative Characteristics of Throwing Movement

**FIGURE 3:**
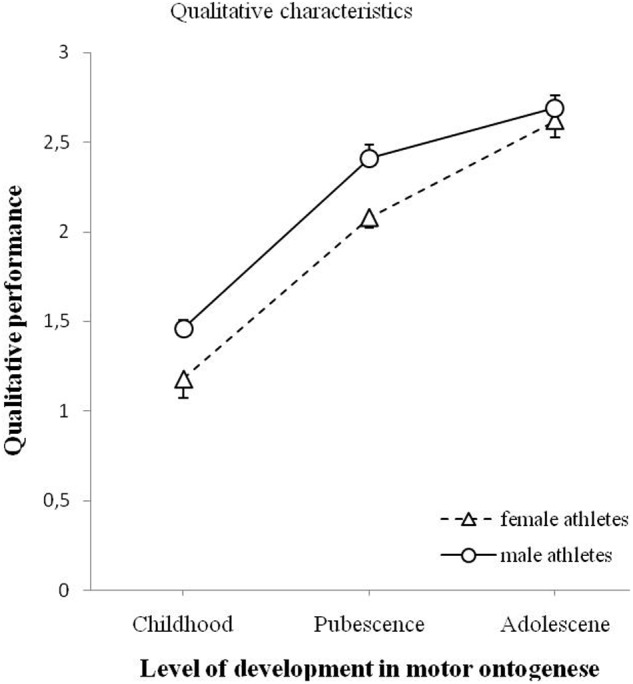
**Qualitative performances for male and female athletes as a function of level of development in motor ontogenesis.** The solid line (circles) illustrates the qualitative performance of male athletes. The dashed line (triangles) illustrates the qualitative performance of female athletes. The qualitative performance was in a range of.0 to 3.0 and was calculated as the mean of three independent expert ratings (cf. Materials and Methods section). Standard error marked by the error bars.

**Figure 3** illustrates the relationship between the qualitative performance for all male and female athletes and the level of development in motor ontogenesis (childhood, pubescence, and adolescence). Using ANOVA, comparisons were made regarding the effect of gender and the effect of age on the qualitative performance and the interaction effect between gender and age on qualitative performance. An ANOVA yielded a significant effect of gender on the qualitative performance, *F*(1,90) = 16.155, *p* < 0.001. The analysis showed a significant effect of age on the qualitative performance, *F*(2,90) = 60.652, *p* < 0.001. The interaction effect was not significant, *F*(2,90) = 0.804, *p* = 0.451.

Separate *t*-tests were performed using Bonferroni Holm correction and an adjusted level of significance (*p* = 0.017) to follow-up the main effect of age. *T*-tests showed a significant effect of age on qualitative performance between childhood and pubescence, *t*(67) = -6.192, *p* < 0.001, between pubescence and adolescence, *t*(49) = -3.277, *p* = 0.002, and between childhood and adolescence, *t*(70) = -9.952, *p* < 0.001.

**Table 2** presents a summary of the variable means and standard derivation of the qualitative performance of male and female athletes in all components (trunk, humerus, forearm, stepping, and backswing actions). An ANOVA was conducted to examine the effect of age and effect of gender for each component separately.

**Table 2:** Variable means and standard deviations of qualitative performance of athletes tracked cross-sectional.

Component	Handball-player			
	
	Female	*SD*	Male	*SD*
**Childhood**				
Trunk action	1.88	0.37	2.14	0.47
Humerus action	1.18	0.44	1.46	0.47
Forearm action	1.37	0.67	1.43	0.48
Stepping	1.59	0.64	2.12	0.51
Backswing	1.96	0.20	1.96	0.23
**Pubescence**				
Trunk action	1.38	0.44	2.13	0.48
Humerus action	2.08	0.49	2.41	0.80
Forearm action	2.75	0.37	2.77	0.47
Stepping	2.08	0.28	2.63	0.41
Backswing	2.05	0.19	2.33	0.34
**Adolescence**				
Trunk action	2.04	0.64	2.63	0.40
Humerus action	2.62	0.73	2.69	0.79
Forearm action	2.89	0.41	2.93	0.20
Stepping	2.52	0.55	2.75	0.33
Backswing	2.33	0.34	2.33	0.33


*Variable means and standard derivation for all components within all groups.*

**FIGURE 4:**
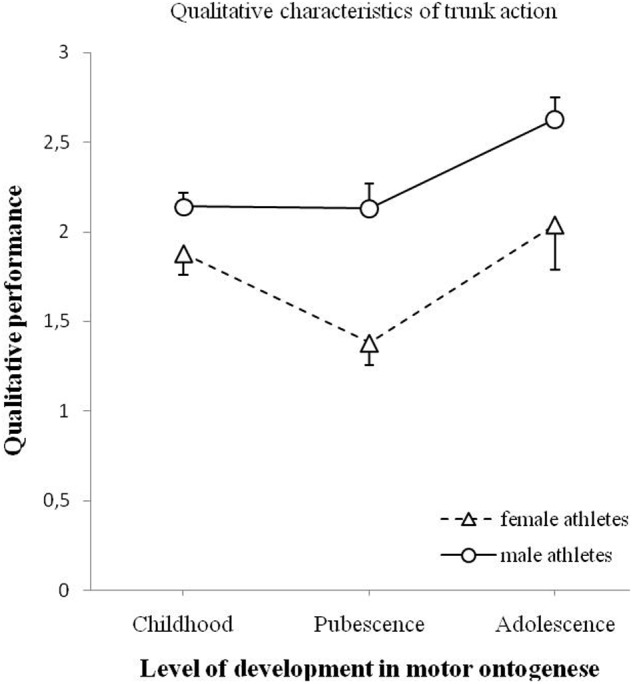
**Changes of qualitative performance of the trunk action (minimum 0 – maximum 3) for male and female athletes as a function of motor ontogenese (childhood, pubescence, and adolescence).** The solid line (circles) illustrates the qualitative performance of male athletes. The dashed line (triangles) illustrates the qualitative performance of female athletes. Standard error marked by the error bars.

**Figure 4** shows the changes in the quality of trunk action for male and female athletes. The main effect of age was significant, *F*(2,90) = 8.700, *p* < 0.001. The main effect of gender was also significant, *F*(1,90) = 23.388, *p* < 0.001. The interaction effect of age and gender on trunk action was not significant, *F*(2,90) = 1.759, *p* = 0.178.

**FIGURE 5:**
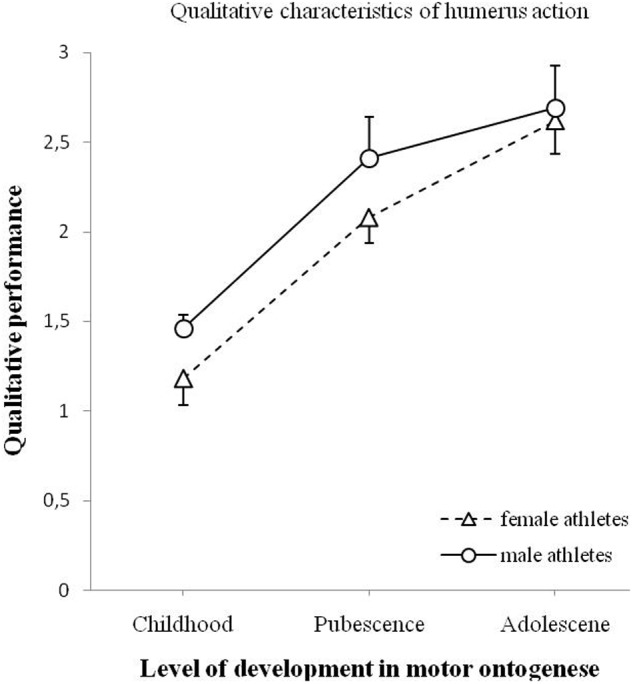
**Changes of qualitative performance of the humerus action (minimum 0 – maximum 3) for male and female athletes as a function of motor ontogenese (childhood, pubescence, and adolescence).** The solid line (circles) illustrates the qualitative performance of male athletes. The dashed line (triangles) illustrates the qualitative performance of female athletes. Standard error marked by the error bars.

**Figure 5** shows the changes in the quality of humerus action. The main effect of age show a significant influence, *F*(2,90) = 36.136, *p* < 0.001. The effect of gender was not significant, *F*(1,90) = 2.840, *p* = 0.095. The analysis showed no significant interaction effect between age and gender on humerus action, *F*(2,90) = 0.340, *p* = 0.713.

**FIGURE 6:**
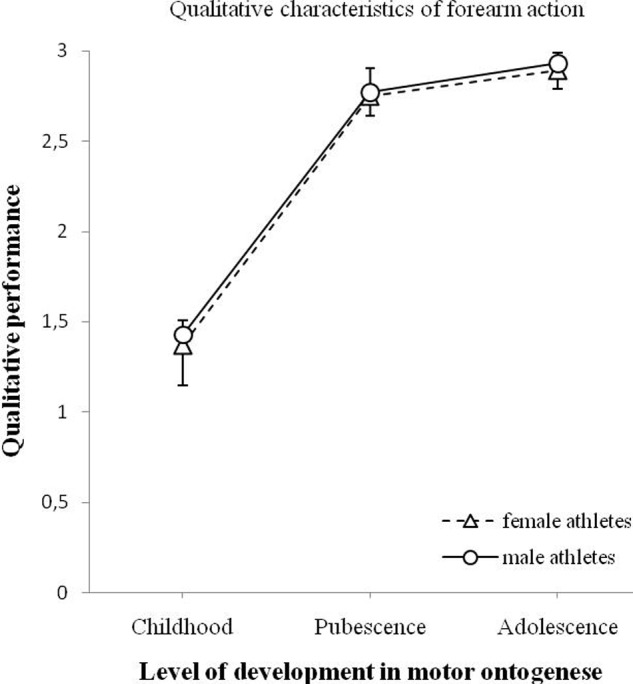
**Changes of qualitative performance of the forearm action (minimum 0 – maximum 3) for male and female athletes as a function of motor ontogenese (childhood, pubescence, and adolescence).** The solid line (circles) illustrates the qualitative performance of male athletes. The dashed line (triangles) illustrates the qualitative performance of female athletes. Standard error marked by the error bars.

**Figure 6** shows the changes in the quality of forearm action for male and female athletes. A significant effect of age on the quality of forearm action was found, *F*(2,90) = 90.895, *p* < 0.001. There was no significant effect of gender on the movement quality of the forearm, *F*(1,90) = 0.192, *p* = 0.662. The interaction between age and gender on the quality of forearm action was not significant, *F*(2,90) = 0.011, *p* = 0.989.

**FIGURE 7:**
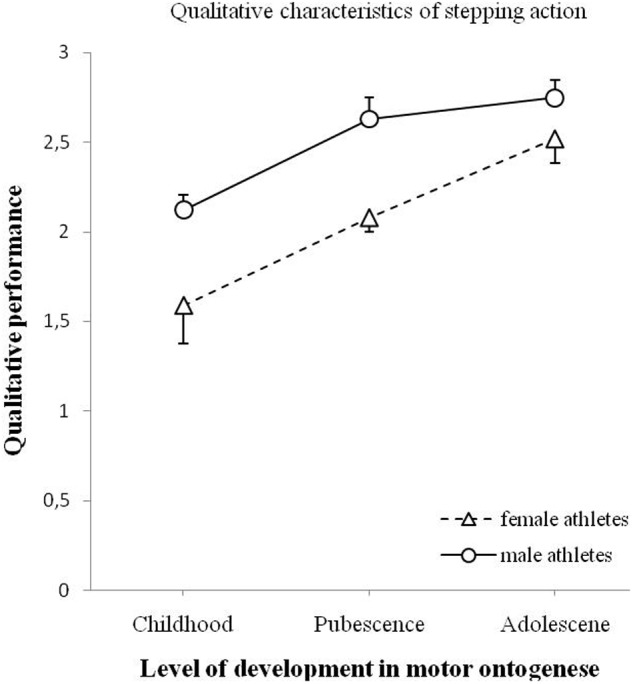
**Changes of qualitative performance of the stepping action (minimum 0 – maximum 3) for male and female athletes as a function of motor ontogenese (childhood, pubescence, and adolescence).** The solid line (circles) illustrates the qualitative performance of male athletes. The dashed line (triangles) illustrates the qualitative performance of female athletes. Standard error marked by the error bars.

The changes in the quality of stepping for male and female athletes are visualized in **Figure 7**. The ANOVA yielded a significant effect of age on the quality of stepping action, *F*(2,90) = 18.581, *p* < 0.001. The analysis showed a significant effect of gender on the quality of stepping, *F*(1,90) = 16.259, *p* < 0.001. An interaction effect of age and gender was not significant, *F*(2,90) = 0.867, *p* = 0.424.

**FIGURE 8:**
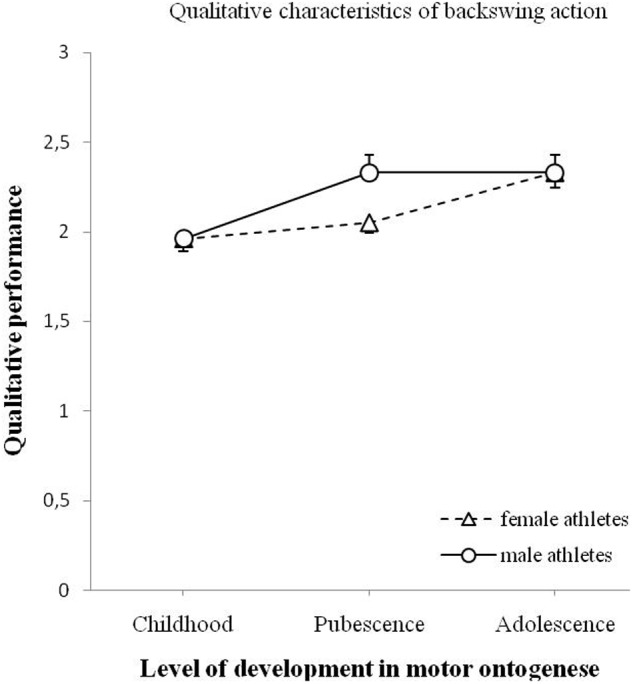
**Changes of qualitative performance of the backswing action (minimum 0 – maximum 3) for male and female athletes as a function of motor ontogenese (childhood, pubescence, and adolescence).** The solid line (circles) illustrates the qualitative performance of male athletes. The dashed line (triangles) illustrates the qualitative performance of female athletes. Standard error marked by the error bars.

The changes in the quality of backswing action are shown in **Figure 8**. The analysis indicated a significant effect of age on the quality of backswing action, *F*(2,90) = 12.576, *p* < 0.001. The analysis showed no significant effect of gender on the quality of backswing action, *F*(1,90) = 2.192, *p* = 0.142. An interaction effect of age and gender was not significant, *F*(2,90) = 2.100, *p* = 0.128.

**Table 3** demonstrates effects of gender in qualitative performance of all components using *t*-tests for independent samples. Gender differences in favor of males were found for trunk, stepping, and backswing actions. The results showed that there are significant differences in trunk action within pubescence (*p* = 0.001) and adolescence (*p* = 0.007). Throughout the three different levels of development in the motor ontogenesis different moving patterns can be observed in stepping action and backswing action. A significant difference was found for stepping action within childhood (*p* = 0.012) and pubescence (*p* = 0.001) and for backswing action within pubescence (*p* = 0.027). Throughout the different levels of development in the motor ontogenesis, no gender differences can be seen in the development of humerus and forearm actions.

**Table 3:** *T*-tests for independent samples showed effects of gender on five components (trunk, humerus, forearm, stepping, and backswing actions) according to levels of development in the motor ontogenesis (childhood, pubescence, and adolescence).

Component	*t* (df)		*t* (df)		*t* (df)	
	
	Childhood		Pubescence		Adolescence	
(1) Trunk	-1.522 (43)	*p* = 0.135	-3.961 (22)	*p* = 0.001	-2.942 (24.897)	*p* = 0.007
(2) Humerus	-1.574 (43)	*p* = 0.123	-1.221 (18.268)	*p* = 0.238	-0.257 (25)	*p* = 0.799
(3) Forearm	-0.331 (43)	*p* = 0.742	-0.158 (22)	*p* = 0.876	-0.321 (25)	*p* = 0.751
(4) Stepping	-2.634 (43)	*p* = 0.012	-3.817 (22)	*p* = 0.001	-1.374 (24.703)	*p* = 0.182
(5) Backswing	0.000 (43)	*p* = 1.000	-2.419 (17.148)	*p* = 0.027	0.000 (25)	*p* = 1.000


In order to compare the development of all movement components, *t*-tests for independent samples were used. **Tables 4** and **5** illustrate effects of age in qualitative performance for male and female athletes.

**Table 4:** *T*-tests for independent samples showed effects of age (childhood to pubescence, childhood to adolescence, and pubescence to adolescence) on five components (trunk, humerus, forearm, stepping, and backswing actions) involving male athletes.

Component	*t* (df)					
	
	Childhood – Pubescence		Childhood – Adolescence		Pubescence – Adolescence	
(1) Trunk	0.058 (46)	*p* = 0.954	-3.079 (45)	*p* = 0.004	-2.664 (21)	*p* = 0.015
(2) Humerus	-3.878 (13.696)	*p* = 0.002	-6.708 (45)	*p* < 0.001	-0.892 (21)	*p* = 0.383
(3) Forearm	-8.351 (46)	*p* < 0.001	-14.918 (40.472)	*p* < 0.001	-1.038 (21)	*p* = 0.311
(4) Stepping	-3.177 (46)	*p* = 0.003	-3.870 (45)	*p* < 0.001	-0.751 (21)	*p* = 0.461
(5) Backswing	-3.432 (14.523)	*p* = 0.004	-3.432 (13.211)	*p* = 0.004	0.000 (21)	*p* = 1.000


**Table 5:** *T*-tests for independent samples showed effects of age (childhood to pubescence, childhood to adolescence, and pubescence to adolescence) on five components (trunk, humerus, forearm, stepping, and backswing actions) involving female athletes.

Component	*t* (df)					
	
	Childhood – Pubescence		Childhood – Adolescence		Pubescence – Adolescence	
(1) Trunk	-2.722 (19)	*p* = 0.014	-0.752 (22.930)	*p* = 0.460	-3.011 (26)	*p* = 0.006
(2) Humerus	-4.294 (19)	*p* < 0.001	-5.362 (23)	*p* < 0.001	-2.215 (26)	*p* = 0.036
(3) Forearm	-5.959 (19)	*p* < 0.001	-7.018 (23)	*p* < 0.001	-0.952 (26)	*p* = 0.350
(4) Stepping	-2.141 (10.445)	*p* = 0.057	-3.791 (23)	*p* = 0.001	-2.695 (23.542)	*p* = 0.013
(5) Backswing	-1.072 (19)	*p* = 0.297	3.400 (22.921)	*p* = 0.002	-2.712 (24.342)	*p* = 0.012


The basis for overall development in throwing movements based on purposeful coaching within the childhood age band. The largest learning progress can be observed in young male and female athletes between childhood and pubescence age bands, with two exceptions: Male athletes did not develop with regard to trunk action; and female athletes did not develop with regard to stepping and backswing action. In total, both groups show the largest development between childhood and adolescence. Only female athletes did not develop their trunk action between these two levels (*p* = 0.460). Regarding the development between pubescence and adolescence, the findings indicate only low development within male athletes. This group had already reached a high level in pubescence, and show stagnation until adolescence. The differences between pubescence and adolescence are not significant, except for trunk action (*p* = 0.015). Contrary to males, the development of female athletes is significant in all components, with exception to forearm action. Forearm action and humerus action were shown already at a high level of development.

## Discussion

This cross-sectional study analyzed the development of the qualitative and quantitative throwing performance of male and female athletes (aged 6 to 16 years). The aim of this study was to examine whether or not the quantitative and qualitative throwing performance of females and males differ. Furthermore the authors explored whether all components are equally affected by gender differences.

### Quantitative Characteristics of Throwing Movement

At first, the quantitative characteristics of throwing movement were examined. It was hypothesized that the quantitative performance of the overarm throwing movement would differ between male and female athletes. This hypothesis can be rejected. It has been shown that there are no significant differences in the quantitative performance of female and male handball athletes. Even in childhood age band, no gender differences can be seen. These results contradict many previous studies that document comparisons of novices and experts alike (e.g., Keogh, 1969; Vogt, 1978; Thomas and French, 1985; van den Tillaar and Ettema, 2004; Ahnert and Schneider, 2007; Rousanoglou et al., 2015). These studies consistently point to a better quantitative performance in the throwing behavior in favor of males. Some indicate that only male performance increases, while female performance levels off (Roberton and Konczak, 2001). Within a 2-year, nominal differences in quantitative throwing performances are possibly compensated by specific exercises and coordinated training.

### Qualitative Characteristics of Throwing Movement

It had also been hypothesized that the quality of the overarm throwing movement differs between male and female athletes. This hypothesis can be accepted, but must be considered in more detail. A significant gender difference can only be seen in the pubescence (*p* < 0.001). This finding can attest statements made by Meinel and Schnabel (1998). They state that there is high growth rate in quality and quantity throwing performances of male in the pubescence, whereas female only “show small jumps in development and only insufficient quality of movement” (Meinel and Schnabel, 1998, p. 317). Sometimes the performance level of female adolescents stagnates. Until adolescents, female participants can compensate for these differences. This also conforms to results found by Lorson et al. (2013). They found significant gender differences only within the adult group for the humerus action, and also speak of a narrowing gender gap in component levels. When designing the present study, the authors assumed that there were gender based qualitative and quantitative differences in throwing performance of young athletes. Surprisingly, there are gender differences in the qualitative performances of the throwing movements, but not in the quantitative performances of the throwing movements. A faulty throwing movement technique can lead to similar quantitative performances such as seen in a correct throwing movement technique. This could be due to the fact that major movement characteristics are developed. Therefore below it is discussed how single components of the overarm throwing movement are influenced by gender differences.

From 6 to 16 years of age, female athletes demonstrated the same moving pattern in trunk action. They demonstrate a block rotation, and did not show development in that movement pattern. The male athletes showed a moderate quality of movement in the trunk action, too. At the adolescence level, male athletes were able to throw with a more differentiated trunk rotation, which dissolves from the bottom up, whereas female athletes still demonstrate a block rotation. Concerning the trunk action, present results probably indicate that female athletes may have difficulties in achieving a well-coordinated movement pattern in trunk action. This finding can be attested by study statements made by Nelson et al. (1991), Menzel (1999), and Goodway and Lorson (2008). The forearm whip is one of the most important nodes in the overarm throwing movement. This movement is composed of a combination of the humerus and forearm actions. From 12 to 16 years of age, a constant development within male and female athletes can be recognized. That leads to a correct forearm lag with a humerus action over shoulder height. Within the childhood and the pubescence the stepping action of male and female athletes were different. Male athletes showed significantly better qualitative movement in stepping action. Female athletes mostly showed an ipsilateral step and a contralateral long step. Male athletes mainly demonstrated a contralateral step. In adolescence, gender differences are compensated. Male and female athletes show different developmental progressions in backswing action, which is in a line with Wagner et al. (2012). During childhood, both showed similar throwing patterns in backswing action. In the pubescence, the male athletes developed significantly, whereas the female athletes stagnated at the level of childhood. From pubescence to adolescence, only the females improved their throwing patterns in backswing action. Male athletes, on the other hand, stagnated at the level of pubescence. During adolescence, male and female athletes perform the backswing in the same way.

From a dynamical systems perspective in which a large number of factors determined the complexity of movement, we may have to rethink the idea of common throwing techniques, in particular as regards the development of throwing techniques. In fact, in real game situations or other competitions similar performances are often the results of different motor strategies (Bauer and Schöllhorn, 1997; Preatoni, 2010). In the case of our study, the disparate development of throwing components between male and female athletes may be justified by this non-linear point of view. Wagner et al. (2011) analyzed the movement variability in various throwing techniques and skill levels in team-handball. Results indicated a decrease in movement variability in skilled and highly skilled handball athletes. Furthermore, Button et al. (2003) analyzed the variability of basketball free-throw action and found that improvement in performance level was correlated with increased movement variability to the end of the throwing arm.

A closer look at the qualitative movement of each component shows that male and female athletes demonstrated different movement patterns in trunk action throughout all age bands. Within the childhood and the pubescence there are also differences in the course of development in backswing and stepping actions. From a functional point of view, it is important to see that the main function phase (humerus and forearm actions) of the throwing movement is not affected by gender differences throughout the different age bands. The present results show that only the secondary and primary auxiliary functional phases (stepping, trunk, and backswing actions) are affected. The aim of preparatory phases is to optimize the main function phase, which is directly related to the overall action goal (Göhner, 1992). In game situations, in which handball athletes respond quickly and use opportunities to throw, there is very little time for placing the right stepping position or managing a large backswing. To be successful in a throw on goal, in contrast to the main function phase, these auxiliary functional phases play a subordinate role. In addition, the main function phase of the overarm throwing movement is identical to main function phases of various kinds of throwing which are used in different game situations, e.g., jump throw or throwing with run-up. This indicates that the main functional phase is well established in motor skills of athletes. From a biomechanical perspective throwing, kicking and striking movements are characterized by the principle of temporal coordination of single components, which is a proximal-to-distal linkage system. The particular difficulty of throwing movements is the optimal coordination of body segments (Hochmuth, 1981; Hamilton and Tate, 2002). These statements argue in favor of the cognitive movement representation, in which Basic Action Concepts (BACs) can be associated with main phase or auxiliary phases and biomechanical aspects (Göhner, 1992; Schack, 2012).

## Conclusion

In this study, it can be seen, that with increasing age, qualitative and quantitative performance of male and female athletes improves. The results suggest that there are gender-specific differences in qualitative throwing performance, but not necessarily in quantitative throwing performance. A closer look shows that differences in the qualitative throwing performance can only be seen in specific components: trunk, stepping and backswing actions, and on certain levels of development. Male and female athletes demonstrated similar movement patterns in humerus and forearm actions. The forearm and humerus actions form the forearm whip, which is the main function phase and the most important component in the overarm throwing movement. Looking to the performance, we recognized that male and female handball athletes lead to a proficient mover, which in turn speaks for a purposeful intervention. In addition, it is very important to see that developmental differences are apparently not immutable. The trunk and the stepping actions represent main faults in the development of the throwing movement for female athletes. The main fault amongst male athletes is also the trunk action.

Although the results were cross-sectional, this data highlights the possibility to compensate deficits in throwing performance. A specific training can apparently alter the throwing pattern of males and females and improve the quantitative and qualitative throwing outcome, which contradicts the findings of Thomas et al. (1994). The representation of gender differences in some publications provoked prejudices and gender-stereotypes, and thus, the expectations in different sports and disciplines (Alfermann, 2009, p. 3). Training intervention and school and university education should equally encourage and support both sexes in learning complex motor skills, such as throwing movements. Also, as Menzel (1999) pointed out, theory and practice should refer to the respective developmental level of children and adolescents which should then lead to individualized instructions.

These results are limited by the controlled setting in which the testing was performed. In this well-defined environment, there were no typical game situations, such as action of opponents, tactical guidance, temporal and spatial pressure situations, or psychological pressure by the audience. The participants could concentrate on their attempts with little disturbing influence from the outside. Obviously, the throwing situation that was conducted in this study is very different from situations in team-handball competitions. It is possible that throwing techniques in a controlled setting may be different in field settings or real game situations. It remains to be shown, how far fundamental motor skills can be transferred to game situations. This issue is similar to recent studies (van den Tillaar and Ettema, 2004, 2006; Wagner et al., 2011, 2012). Whereas Lorson (2003) suggested that body component levels for the step, trunk, and forearm actions demonstrated in a real game situation were correlated with the body component levels demonstrated during practice. Therefore subsequent studies could be conducted in team-handball competitions in a longitudinal design. This may provide valuable insights, but need also many resources. Explorations of the present study in practice situations and control situations in physical education in the schools are entirely conceivable. For individualized coaching (Menzel, 1999), further research should also focus on cognitive measurements of throwing movements. The Dimension Analysis-Motoric diagnostics (Schack, 2012) could provide a possible approach that helps to assess the cognitive movement representations of function phase, and their role for accuracy of movement execution.

## Author Contributions

MG, DK, and TS developed and designed the study. MG collected the data. MG and DK analysed the data. MG, DK, and TS interpreted the findings and wrote up the manuscript.

## Conflict of Interest Statement

The authors declare that the research was conducted in the absence of any commercial or financial relationships that could be construed as a potential conflict of interest.

## References

[B1] Ahnert J. Schneider W. (2007). Entwicklung und stabilität motorischer fähigkeiten vom vorschul- bis ins frühe erwachsenenalter. Befunde der münchner längsschnittstudie LOGIK. *Z. Entwicklungspsychol. Pädagog. Psychol.* 39 12–24. 10.1026/0049-8637.39.1.12

[B2] Alfermann D. (2009). “Geschlechtstypik der motorischen Entwicklung,” in *Handbuch Motorische Entwicklung*, eds Baur J. Bös K. Conzelmann A. Singer R. (Schorndorf: Hofmann), 251–260.

[B3] Barrett D. D. Burton A. W. (2002). Throwing patterns used by collegiate baseball players in actual games. *Res. Q. Exerc. Sport* 73 19–27. 10.1080/02701367.2002.10608988 11926482

[B4] Bauer H. U. Schöllhorn W. (1997). Self-organizing maps for the analysis of complex movement patterns. *Neural Process. Lett.* 5 193–199. 10.1023/A:1009646811510

[B5] Bingham G. P. Schmidt R. C. Rosenblum L. D. (1989). Hefting for a maximum distance throw: a smart perceptual mechanism. *J. Exp. Psychol. Hum. Percept. Perform.* 15 507–528. 10.1037/0096-1523.15.3.507 2527959

[B6] Burton A. W. Rodgerson R. W. (2003). “Development of throwing,” in *Development of Movement Coordination in Children. Applications in the Fields of Ergonomics, Health Sciences and Sport*, eds Savelsbergh G. Davids K. van der Kamp J. Bennett S. (London: Routledge).

[B7] Button C. MacLeod M. Sanders R. Coleman S. (2003). Examining movement variability in the basketball free throw action at different skill levels. *Res. Q. Exerc. Sport* 74 257–269. 10.1080/02701367.2003.10609090 14510290

[B8] Ehl T. Langendorfer S. J. Roberton M. A. (2005). Does the throwing “Gender gap” occur in Germany? *Res. Q. Exerc. Sport* 76 488–493. 10.1080/02701367.2005.10599322 16739686

[B9] Etnyre B. R. (1998). Accuracy characteristics of throwing as a result of maximum force effort. *Percept. Mot. Skills* 86 1211–1217. 10.2466/pms.1998.86.3c.1211 9700796

[B10] Göhner U. (1992). *Einführung in Die Bewegungslehre des Sports, Teil 1: Die Sportlichen Bewegungen.[Introduction into Teaching Movement Science and Sport, Part I: The Athletic Movement].* Schorndorf: Verlag Karl Hoffmann.

[B11] Goodway J. D. Lorson K. M. (2008). Gender differences in throwing form of children ages 6-8 years during a throwing game. *Res. Q. Exerc. Sport* 79 174–182. 10.1080/02701367.2008.10599481 18664042

[B12] Gorostiaga E. M. Granados C. Ibáñez J. Izquierdo M. (2005). Differences in physical fitness and throwing velocity among elite and amateur male handball players. *Int. J. Sports Med.* 26 225–232. 10.1055/s-2004-820974 15776339

[B13] Halverson L. E. Roberton M. A. (1984). *Developing Children - their Changing Movement: A Guide for Teachers.* Philadelphia, PA: Lea & Febiger.

[B14] Halverson L. E. Roberton M. A. Langendorfer S. (1982). Development of the overarm throw: movement and ball velocity changes by seventh grade. *Res. Q. Exerc. Sport* 53 198–205. 10.1080/02701367.1982.10609340

[B15] Hamilton M. L. Tate A. L. (2002). “Constraints on throwing behavior: an exploratory analysis,” in *Advances in Motor Development Research American Alliance for Health, Physical Education, Recreation, and Dance* Vol. 2 ed. Clark J. E. (Reston, VA: NASPE Publications), 49–61.

[B16] Hochmuth G. (1981). *Biomechanik Sportlicher Bewegungen.(2. Auflage).* Frankfurt: Limpert.

[B17] Holm S. (1979). A simple sequentially rejective multiple test procedure. *Scand. J. Stat.* 6 65–70.

[B18] Intermill C. Husak W. S. (1984). Relationship between speed and accuracy in an over-arm throw. *Percept. Mot. Skills* 59 219–222. 10.2466/pms.1984.59.1.219

[B19] Karadenizlil Z. I. Inal H. S. Meriç B. Aydin M. Bulgan Ç (2014). Accuracy and velocity of the elite female turkish handball players. *Int. J. Sports Sci.* 4 21–26. 10.5923/j.sports.20140401.04

[B20] Keogh J. F. (1969). *Change in Motor Performance During Early School Years.* Los Angeles, CA: Department of Physical Education, University of California.

[B21] Kolodziej C. (2010). *Richtig Handball Spielen*, 3rd Edn München: BLV Verlagsgesellschaft.

[B22] Langendorfer S. J. Roberton M. A. (2002). Individual pathways in the development of forceful throwing. *Res. Q. Exerc. Sport* 73 245–256. 10.1080/02701367.2002.10609018 12230331

[B23] Lorson K. M. (2003). *The Influence of Three Instructional Strategies on the Performance of the Overarm Throw.* Doctoral dissertation, Ohio State University, Columbus, OH.

[B24] Lorson K. M. Stodden D. F. Langendorfer S. J. Goodway J. D. (2013). Age and gender differences in adolescent and adult overarm throwing. *Res. Q. Exerc. Sport* 84 239–244. 10.1080/02701367.2013.784841 23930550

[B25] Meinel K. Schnabel G. (1998). *Bewegungslehre – Sportmotorik: Abriß einer Theorie der Sportlichen Motorik Unter Pädagogischem Aspekt.* Berlin: Sportverlag.

[B26] Menzel H. J. (1999). *Inter-individual Differences of Movement Patterns in Javelin Throw.* Calgary: ISB, 126.

[B27] Morris A. Williams J. Atwater A. Wilmore J. (1982). Age and sex differences in motor performance of 3 through 6 year old children. *Res. Q. Exerc. Sport* 53 214–221. 10.1080/02701367.1982.10609342

[B28] Nelson R. N. Thomas J. R. Nelson J. K. (1991). Longitudinal change in throwing performance. *Res. Q. Exerc. Sport* 62 105–108. 10.1080/02701367.1991.10607526 2028085

[B29] Preatoni E. (2010). Motor variability and skills monitoring in sports. *Paper Presented at the XXVIII International Conference on Biomechanics in 1042 Sports Marquette*, Milwaukee, WI.

[B30] Rivilla-García J. Navarro Valdivielso F. Grande Rodriguez I. Ignatova A. S. Sampedro Molinuevo J. (2010). Differences in throwing capacity between senior and U-18 men handball players. *Serbian J. Sport Sci.* 4 145–151.

[B31] Roberton M. A. Konczak J. (2001). Predicting children’s overarm throw ball velocities from their developmental levels in throwing. *Res. Q. Exerc. Sport* 72 91–103. 10.1080/02701367.2001.10608939 11393884

[B32] Roth K. Kröger C. Memmert D. (2002). *Ballschule Rückschlagspiele.* Schorndorf: Hofmann.

[B33] Rousanoglou E. N. Noutsos K. S. Bayios I. A. Boudolos K. D. (2015). Self-Paced and temporally constrained throwing performance by team-handball experts and novices without foreknowledge of target position. *J. Sports Sci. Med.* 14 41–46. 25729288 PMC4306781

[B34] Schack T. (2012). “Measuring mental representations,” in *Measurement in Sport and Exercise Psychology*, eds Tenenbaum G. Eklund R. C. Kamata A. (Champaign, IL: Human Kinetics), 203–214.

[B35] Schack T. Bar-Eli M. (2007). “Psychological factors in technical preparation,” in *Psychology of Sport Training*, eds Blu-menstein B. Lidor R. (Münster: Meyer & Meyer), 62–103.

[B36] Schott N. (2010). “Entwicklung des Werfens,” in *Motorische Entwicklung*, eds Schott N. Munzert J. (Göttingen: Hogrefe), 127–148.

[B37] Thomas J. R. French E. (1985). Gender differences across age in motor performance – a meta-analysis. *Psychol. Bull.* 98 269–282. 10.1037/0033-2909.98.2.260 3901062

[B38] Thomas J. R. Michael D. Gallagher J. D. (1994). Effects of training on gender differences in overhand throwing: a brief quantitative literature review. *Res. Q. Exerc. Sport* 65 67–71. 10.1080/02701367.1994.10762209 8184213

[B39] van den Tillaar R. Ettema G. (2003). Influence of instruction on velocity and accuracy of overarm throwing. *Percept. Mot. Skills* 96 423–434. 10.2466/PMS.96.2.423-434 12776824

[B40] van den Tillaar R. Ettema G. (2004). A force-velocity relationship and coordination patterns in overarm throwing. *J. Sports Sci. Med.* 3 211–219. 24624005 PMC3938059

[B41] van den Tillaar R. Ettema G. (2006). A comparison between novices and experts of the velocity-accuracy trade-off in overarm throwing. *Percept. Mot. Skills* 103 503–514. 10.2466/pms.103.2.503-514 17165415

[B42] Vogt U. (1978). *Die Motorik 3- bis 6-jähriger Kinder. Ihre Abhängigkeit vom Biologischen Entwicklungsstand und Sozialen Umweltfaktoren, Beiträge zur Lehre und Forschung im Sport.* Schorndorf: Hofmann.

[B43] Wagner H. Pfusterschmied J. Klous M. van Duvillard S. P. Müller E. (2012). Movement variability and skill level of various throwing techniques. *Hum. Mov. Sci.* 31 78–90. 10.1016/j.humov.2011.05.005 21835479

[B44] Wagner H. Pfusterschmied J. van Duvillard S. P. Müller E. (2011). Performance and kinematics of various throwing techniques in team-handball. *J. Sport Sci. Med.* 10 73–80. PMC3737895 24149298

[B45] Wiener M. (2011). Techniktraining in schule und verein. Heute: die grundwurfarten im handball. *Sportpraxis* 52 19–23.

[B46] World Medical Association [WMA] (2013). World medical association declaration of helsinki ethical principles for medical research involving human subjects. *J. Am. Med. Assoc.* 310 2191–2194. 10.1001/jama.2013.281053 24141714

